# Efficacy, tolerability and barriers to the use of anti-CGRP medications among migraine patients in Egypt: real world experience

**DOI:** 10.3389/fneur.2026.1830276

**Published:** 2026-06-18

**Authors:** Manar Abdelazeem Gomaa, Nourhan Abdelmohsen Taha, Shahinaz Mohamed Helmy, Sherien Mohamed Farag, Ramez Reda Moustafa

**Affiliations:** Department of Neurology, Ain Shams University, Cairo, Egypt

**Keywords:** anti-CGRP, Egypt, migraine, real-world, tolerability, treatment efficacy

## Abstract

**Background:**

With the introduction of anti-CGRP therapies in Egypt in 2019, there is a growing need to evaluate their real-world use, including effectiveness, tolerability, barriers to access, and treatment adherence among migraine patients. This study aimed to describe the clinical outcomes, tolerability, and barriers to the use of anti-CGRP therapies in an Egyptian cohort.

**Methods:**

In this descriptive observational study, migraine patients who were prescribed anti-CGRP therapy were assessed using headache diaries, MIDAS, and HIT-6 at baseline, with follow-up at one and three months after treatment initiation. Patients were also evaluated for background headache and medication overuse headache pre- and post-treatment, the reasons for treatment discontinuation, and relapse rate after drug discontinuation (defined as loss of ≥50% of initial improvement).

**Results:**

A total of 80 patients (62 chronic and 18 episodic migraine) received Erenumab, Galcanezumab, or Rimegepant. Overall, 54 patients (68%) showed a favorable ≥50% response, with clinically significant reduction in monthly migraine days, headache severity, duration, HIT-6 and MIDAS scores (*p* < 0.001), as well as prevalence of background headache and medication overuse. Tolerability was generally favorable and treatment discontinuation occurred in 35 patients, primarily due to either satisfactory improvement, lack of improvement or cost, and was associated with relapse in 42.1% of cases.

**Conclusion:**

This study provides real-world insights into the use of anti-CGRP therapies among migraine patients in Egypt, demonstrating consistent clinical improvement and good tolerability. It also highlights important challenges related to treatment access and adherence. Findings should be interpreted in the context of observational design of the study, and further controlled studies are warranted.

## 1 Introduction

Migraine is a chronic, debilitating neurological disorder affecting approximately 14% of the global population (1), ranking as the second leading cause of disability among young women and the fourth overall cause of years lived with disability (YLDs) worldwide, according to the Global Burden of Disease study (GBD) 2021, being particularly significant in low- and middle-income countries, where it has historically been under-recognized and under-treated (2).

The prevalence of migraine in Egypt varies widely, with studies reporting rates between 3.8 and 20.3%, depending on the methodology and population studied (3–5), with females being predominantly affected, especially during their reproductive years, further contributing to reduced productivity and quality of life in a key demographic (6).

One of the key molecules implicated in migraine pathogenesis is calcitonin gene-related peptide (CGRP), a potent vasodilator and neuropeptide released systemically and from trigeminal afferents. Elevated levels of CGRP have been documented during migraine attacks, and intravenous infusion of CGRP has shown to provoke migraine-like headaches in susceptible individuals. These findings led to the development of CGRP-targeted therapies, which represent the first disease-specific pharmacologic treatments for migraine (7, 8).

Clinical trials and real-world studies around the world have demonstrated their effectiveness in reducing migraine frequency, intensity, and disability, with favorable safety profiles and fewer drug interactions (9).

Before anti-CGRPs became available in Egypt in 2019, clinicians faced a lot of unmet needs in migraine management, and with their introduction emerged the need to emphasize their efficacy and explore obstacles facing migraine patients regarding use and adherence to therapy. Understanding how these therapies perform outside the controlled setting of clinical trials will help tailor treatment strategies, inform policy decisions, and improve patient outcomes.

This study’s primary aim was to describe the efficacy, tolerability, and barriers to the use of available anti-CGRP medications among a sample of migraine patients in Egypt, along with patterns of response to treatment and discontinuation.

## 2 Methods

This is a multicenter descriptive observational study involving public and private headache centers in Cairo, mainly Ain shams university hospitals, Neuromed and Saudi German hospitals headache clinics.

This study received ethical approval from the ethical committee of Department of Neurology and Psychiatry, Ain Shams University (FWA 000017585).

We included cases with the diagnosis of either episodic or chronic migraine – according to international classification of headache disorders (10) who were on anti-calcitonin-gene related peptide medications or have had a trial of anti CGRP medications for a minimum duration of three months, without concurrent usage of other preventive treatments.

The sample size was calculated using PASS 11.0 program^1^ to be ≥ 80 patients to achieve 80% power to reject the null hypothesis with a significance level (alpha< of 0.05 using a two sided one-sample t-test). The sample size was calculated based on the expected change in monthly migraine days from baseline, as the primary within-patient outcome.

After obtaining written informed consent from all participants, patients were interviewed for detailed medical history and headache characteristics in accordance with ICHD-3 criteria. All patients completed a semi-structured questionnaire for demographic data, relevant personal and family history, comorbidities, baseline headache characters [frequency/month, duration and headache intensity using the Numerical Pain Rating scale (11)], previous medication trials and duration of use of anti-CGRP therapy, causes of discontinuation of drug and relapse.

Patients were also assessed using the Migraine Disability Assessment Scale (MIDAS) (Arabic version) (12) and the Headache impact test (HIT-6): Arabic version (13) at baseline, with follow-up assessments at one- and three-months post-initiation of anti-CGRP therapy. Patients already on anti-CGRP treatment for at least three months were retrospectively evaluated at corresponding intervals. Reasons for discontinuation of therapy were recorded, along with assessment of relapse (defined as a loss of ≥50% of initial improvement). Patients were also screened for medication overuse headache (MOH) and generalized background headaches (considered as a continuous or near-continuous low-grade headache reported by patients, typically present daily, and often serving as a baseline pain over which migraine attacks may be superimposed, this phenomenon is frequently observed in patients with chronic migraine and/or medication overuse) both before and after treatment.

### 2.1 Endpoints

The primary endpoint was achieving a reduction of more than 50% in monthly migraine days (MMDs) among the study population. Secondary endpoints included clinically meaningful improvements in attack duration, headache intensity, as well as reductions in HIT-6 and MIDAS scores and reduction in prevalence of MOH and generalized background headache among the study population.

### 2.2 Statistical analysis

Statistical analyses were performed using Statistical Package for Social Sciences (IBM SPSS Statistics for Windows, Version 27.0. Armonk, NY: IBM Corp.).

Continuous variables were assessed for normality using the Shapiro–Wilk test. Non-normally distributed variables are reported as median and interquartile range (IQR). Categorical variables are expressed as frequencies and percentages.

The pre- versus post-treatment analysis among the study population were conducted using the Wilcoxon signed-rank test for non-parametric data, and for categorical variables, comparisons were performed using the chi-square test as expected cell counts were not less than 5.

Considering the baseline differences noticed upon stratifying the study population according to the type of anti-CGRP medication, between-group comparisons were limited to descriptive analyses of percentage change from baseline. Percentage change from baseline was calculated as: [(post-treatment value − baseline value)/baseline value × 100] and is reported descriptively.

Given the observational and real-world nature of the study, analyses were primarily descriptive and exploratory, aiming to determine patterns of response, tolerability, and treatment utilization. Results are therefore interpreted with emphasis on magnitude and clinical relevance, rather than reliance on statistical significance alone.

Level of significance was set as *p* > 0.05: Non-significant and *p* < 0.05: Significant.

## 3 Results

### 3.1 Demographics and baseline characteristics

During the study period, 120 patients were eligible, out of which 40 were excluded, either because of refusal to participate (*N* = 15) or loss to follow up during the study (*N* = 25), so a total of 80 cases were included.

The study included 62 patients with diagnosis of chronic migraine and 18 patients with episodic migraine. Data was pooled for analysis to increase statistical power and generalizability.

The cohort’s demographics showed that the mean age of onset of migraine was 21.9 ± 7 years, 80% were females and 50% had a family history of migraine, with low prevalence for special habits such as smoking (3.75%).

The most common associated co-morbidity reported was insomnia 22.5%, followed by depression and anxiety (17.5 and 15% respectively), then hypertension, gastrointestinal disorders, diabetes mellitus and epilepsy (8.75, 7.5, 5, 2.5% respectively). Additionally, 10% of patients reported other medical conditions such as polycystic ovary and hypothyroidism.

Patients exhibited diverse headache symptomatology, with 32.5% experiencing generalized headache pain, while 30% localized to the left side and 20% to the right. The majority (55%) of patients described their headache as pulsating, fewer described the pain as pressure-like, dull aching, burning, or stabbing in character. As for the associated symptoms, photophobia (87.5%) and phonophobia (82.5%) were the most prevalent, followed by nausea and vomiting (71.25%), then cutaneous allodynia reported in 47.5% of patients (Table 1).

**Table 1:** The clinical characteristics of migraine among the study participants.

Categorical variables		
Variable	*N* (total = 80)	%
Location of pain		
Diffuse	26	32.5
Left	24	30.0
Right	16	20.0
Bilateral	14	17.5
Character		
Pulsating	44	55.0
Dull aching	13	16.2
Pressure	11	13.7
Stabbing	7	8.8
Burning	5	6.2
Associated symptoms		
Photophobia	70	87.5
Phonophobia	66	82.5
MOH	60	75.0
Nausea & Vomiting	57	71.3
Generalized background headache	55	68.8
Cutaneous Allodynia	38	47.5
Autonomic symptoms	25	31.3
Vertigo & Dizziness	20	25.0

MOH, Medication overuse headache.

As regards baseline headache diary parameters, median monthly migraine days (MMDs) was 30 days (IQR = 15–30), headache duration was 24 h (IQR = 24–60), and median intensity was 9.5 (IQR = 8.5–10). Medication overuse headache at baseline was reported in 60 patients, and 55 patients reported generalized background headache. The average HIT-6 score was 71 (IQR 66–72), and MIDAS score was 102.5 (IQR 51–164) (Table 2).

**Table 2:** Changes in clinical variables among study population post-treatment.

Study group (*n* = 80)	Baseline Median (IQR)	Follow up Median (IQR)	*p*-value
Headache frequency (days/month)	30 (15–30)	5 (3–17)	<0.001
MIDAS	102.5 (51–164)	30 (6–85)	<0.001
HIT-6	71 (66–72)	59 (47.5–65)	<0.001
Headache severity	9.5 (8.5–10)	5 (4–7)	<0.001
Headache duration (hours)	24 (24–60)	4 (2–24)	<0.001

Test used, Wilcoxon signed rank test; MIDAS, Migraine disability scale; HIT-6, Headache Impact-6; IQR, Interquartile range.

### 3.2 Treatment history

Before initiating anti-CGRP therapy, patients had an average of 2 (IQR = 1–4) preventive treatment failures. The majority of the patients received Erenumab (61.25%, *n* = 49), 17.5% received Galcanezumab (*n* = 14), and 21.25% received Rimegepant (*n* = 17). The average perceived onset of action (improvement from starting the treatment as reported by the patient) for these therapies was 1 week (IQR 0.88–2 weeks), with average duration of treatment on anti CGRP being 3 months (IQR 3–6 months). Insurance coverage was available for 57.5% of patients (*n* = 46).

### 3.3 Treatment effectiveness

**Figure 1:**
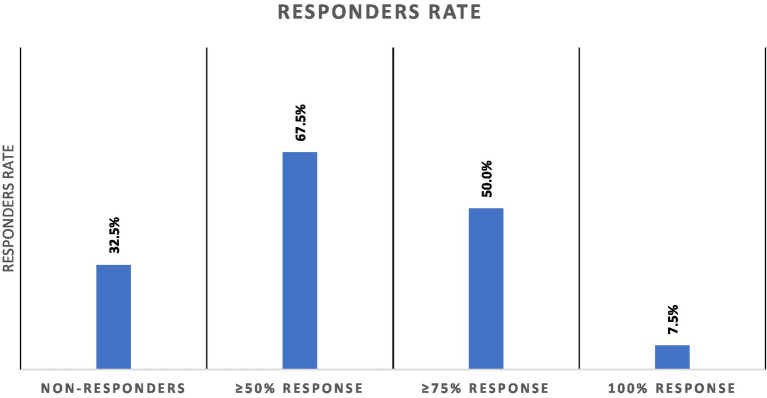
Rate of response among the study population.

**Figure 2:**
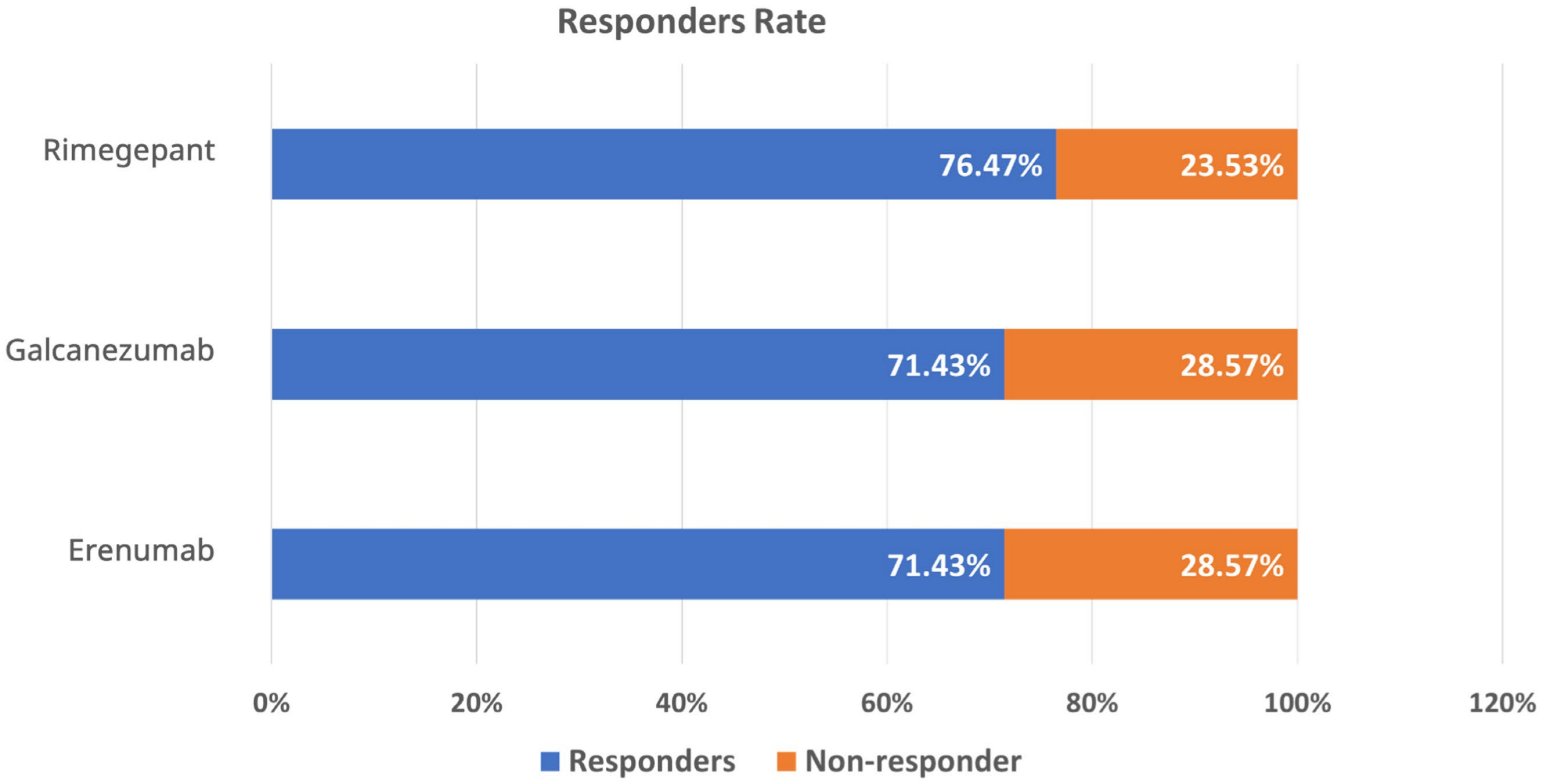
Rate of response for each of the prescribed anti-CGRPs.

Patients were considered responders with a ≥ 50% reduction in MMDs. Overall, the rate of ≥50% response was 68% (54/80) (65% (32/49), 71% (10/14) and 71% (12/17) for Erenumab, Galcanezumab and Rimegepant, respectively) (Figures 1,2).

Analysis of the overall study population demonstrated clinically meaningful improvement across multiple headache-related outcomes following initiation of anti-CGRP therapy, including reductions in MMDs, headache severity and duration, disability scores (MIDAS, HIT-6), prevalence of background headache and MOH (*p* < 0.001) (Tables 2, 3).

**Table 3:** Changes in categorical clinical variables among study population post-treatment.

Study group (*n* = 80)		Baseline	Follow up	*p*-value
		*N* (%)	*N* (%)	
Background generalized headache	No	25 (31.25%)	53 (66.25%)	<0.001
	Yes	55 (68.75%)	27 (33.75%)	
Medication overuse Headache	No	20 (25%)	57 (71.25%)	<0.001
	Yes	60 (75%)	23 (28.75%)	

Test of Significance is McNemar Test.

These differences were also evident when analyzing each medication separately, though no comparisons could be made between groups (Tables 4, 5).

**Table 4:** Percent of change from baseline in clinical outcomes across anti-CGRP treatment groups.

Variable	Erenumab (*n* = 49)	Galcanezumab (*n* = 14)	Rimegepant (*n* = 17)
	Median (IQR)	Median (IQR)	Median (IQR)
% of change in headache frequency	−66.7% (−86.7 to 0%) CI = (−73.3% to −31.3%)	−73.3% (−90% to −33.3%) CI = (−90% to −33.3%)	−83.3% (−90% to −33.3%) CI = (−87.5% to −33.3%)
% of change in MIDAS score	−59.1% (−89.4% to −13.1%) CI = (−76% to −44.4%)	−69.2% (−76.8% to −17%) CI = (−78.6 to 0%)	−85.6% (−89.4% to −51.2%) CI = (−90% to −51.2%)
% of change of HIT-6 score	−15% (−26% to −12%) CI = (−26%% to −5%)	−15% (−25% to −2%)	−31% (−35% to −18%) CI = (−39.0% to −17.0%)
% of change of headache severity	−44.4% (−60% - -30%) CI = (−50% to −37.5%)	−31.7% (−60% - -20%) CI = (−60% to −20%)	−50% (−62.5% to −22.2%) CI = (−55.6% to −22.2%)
% of change of headache duration	−83.3% (−95.8% to −66.7%) CI = (−88.9% to −66.7%)	−66.7% (−83.3 to 100%) CI = (−79.2 to 166.7%)	−66.7% (−94.4% to −50%) CI = (−70% to −25%)

Percentage change was calculated as: (follow-up − baseline)/baseline × 100.

**Table 5:** Changes in clinical outcomes (categorical variables) across anti-CGRP treatment groups at baseline and follow-up.

Variable		Erenumab (*n* = 49)	Galcanezumab (*n* = 14)	Rimegepant (*n* = 17)
		*N* (%)	*N* (%)	*N* (%)
Baseline background generalized headache	No	6 (12.2%)	6 (42.9%)	13 (76.5%)
	Yes	43 (87.8%)	8 (57.1%)	4 (23.5%)
Follow up Background generalized headache	No	44 (89.8%)	3 (21.4%)	6 (35.3%)
	Yes	5 (10.2%)	11 (78.6%)	11 (64.7%)
Within group comparison	*p*	<0.001	0.250	0.016
Baseline Medication overuse	No	7 (14.3%)	4 (28.6%)	9 (52.9%)
	Yes	42 (85.7%)	10 (71.4%)	8 (47.1%)
Follow up Medication overuse	No	41 (83.7%)	7 (50.0%)	9 (52.9%)
	Yes	8 (16.3%)	7 (50.0%)	8 (47.1%)
Within group comparison	*p*	<0.001	0.453	1.00

McNemar test of significance was used for within group comparison.

### 3.4 Tolerability

Among patients receiving Galcanezumab (*n* = 14), three individuals (21.4%) experienced hypertension, fatigue, and insomnia. In the Erenumab group (*n* = 49), 14 patients (28.6%) reported side effects, most commonly constipation (*n* = 8), followed by injection site reactions (*n* = 4), appetite loss (*n* = 1), and blurred vision (*n* = 1). Only one patient (5.9%) in the Rimegepant group (*n* = 7) reported side effects, specifically bony aches.

### 3.5 Discontinuation

**Figure 3:**
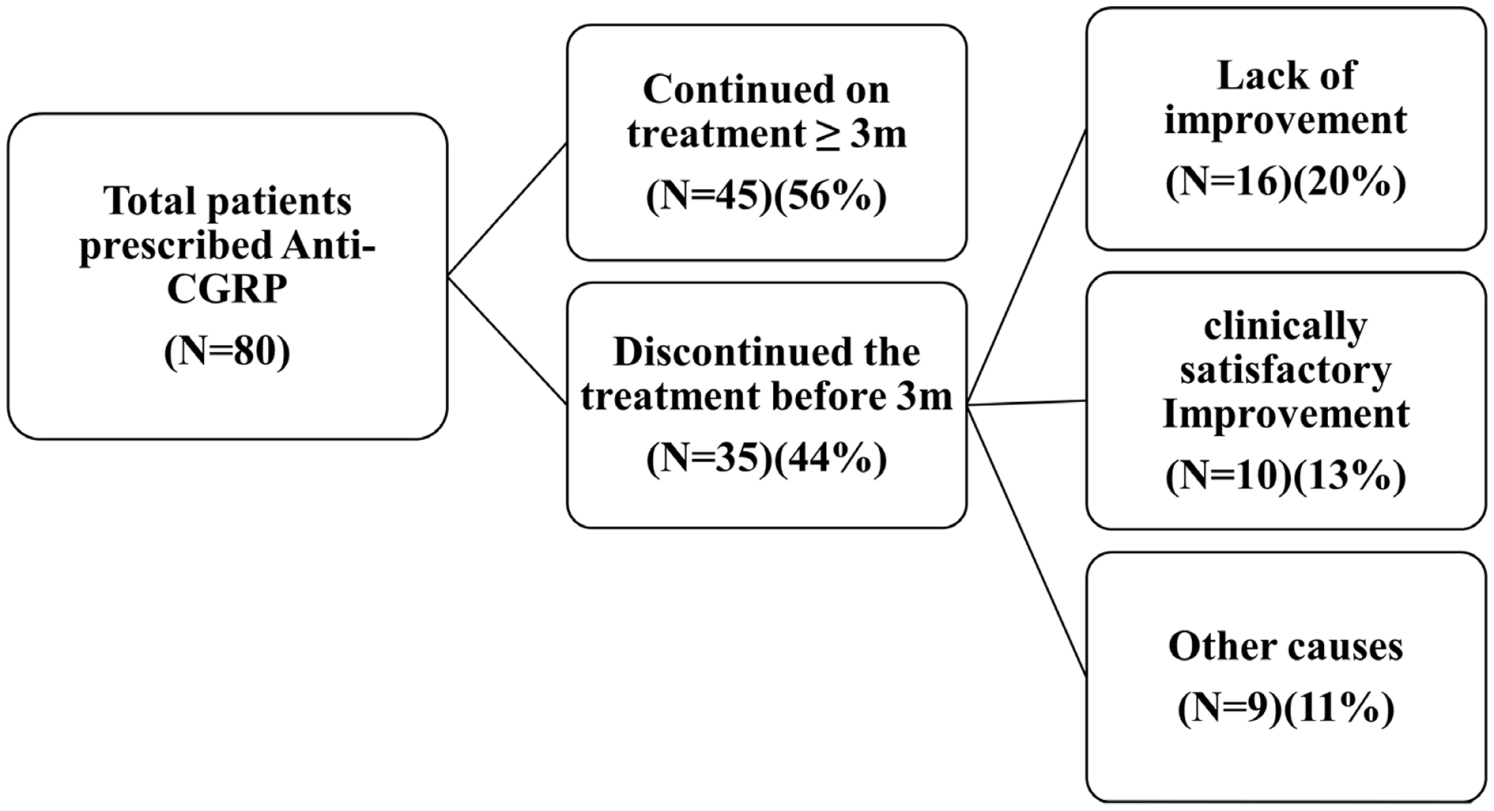
Hierarchy showing discontinuation analysis among study cohort.

**Figure 4:**
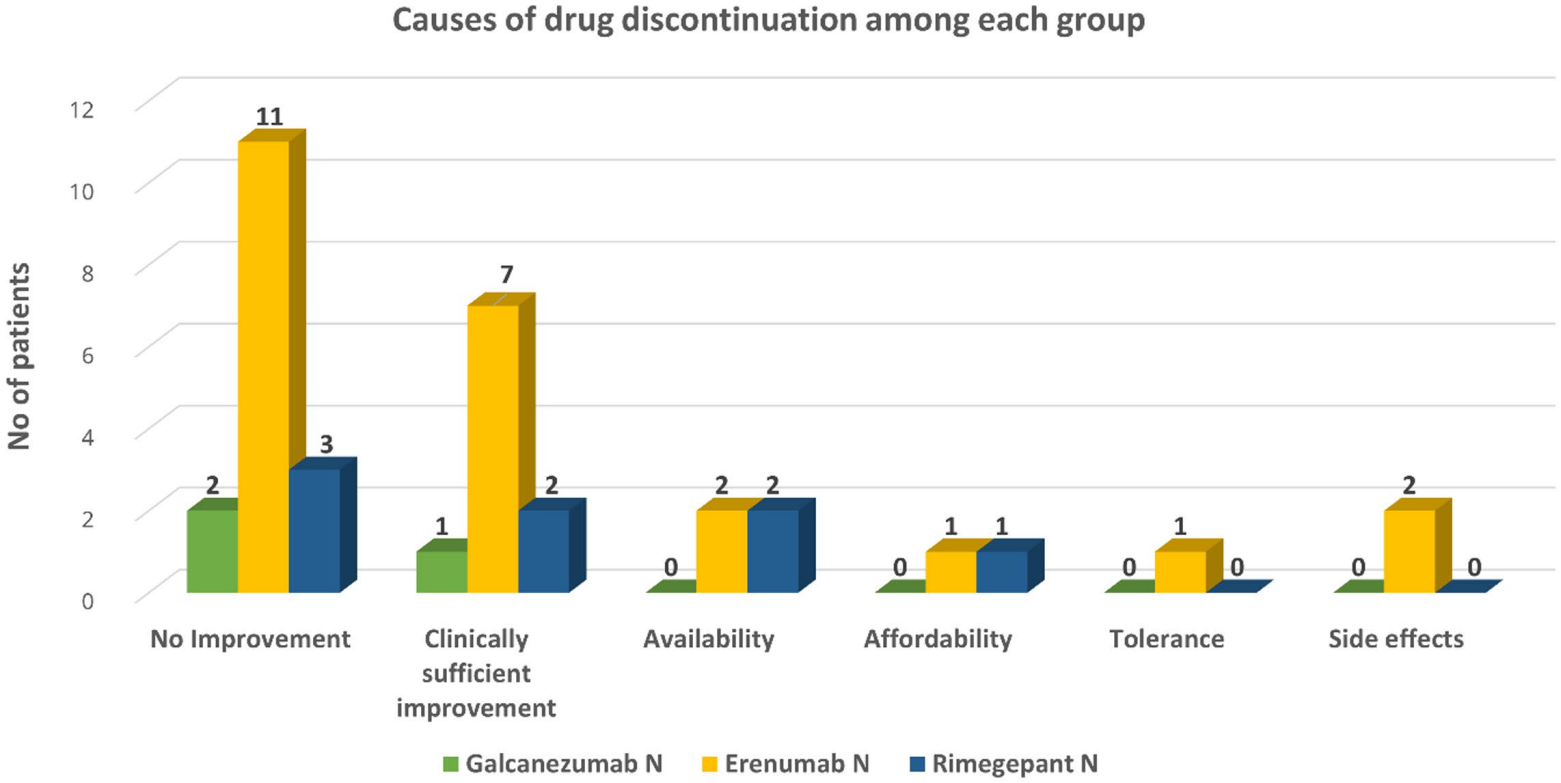
Causes of discontinuation of the anti-CGRP with the prevalence for each cause among the study groups; Galcanezumab (green bars), Erenumab (yellow bars) and Rimegepant (blue bars).

Discontinuation occurred in 35 patients, with the primary reason being lack of improvement (20%), followed by clinically satisfactory improvement (13%). Other factors contributing to discontinuation included difficulty in medication availability, affordability, and side effects (Figures 3, 4).

Regarding relapse, among those who discontinued treatment (*n* = 19), follow-up data was available for 12 patients (6 months at average after discontinuation), of which, 8 patients experienced a recurrence of symptoms, while 4 patients maintained their improvement. Of those who relapsed, seven were in the Erenumab group and one in the Rimegepant group, while no relapses were reported in the Galcanezumab group.

### 3.6 Clinical characteristics and patterns of treatment response

Exploratory analyses of treatment response in relation to patient characteristics—including demographic factors (age, sex, comorbidities, age at onset), headache features (location, character, and associated symptoms), and baseline headache diary parameters (monthly migraine days, attack duration, intensity, analgesic use, and crystal-clear days)—suggested that the presence of medication overuse headache was associated with a lower likelihood of response, although this did not reach statistical significance.

In contrast, factors such as insurance coverage and treatment adherence for at least three months were associated with more favorable response patterns.

Baseline headache frequency, headache severity, and the number of prior preventive treatment failures did not show a clear association with treatment response in this cohort.

These observations should be interpreted cautiously given the observational design and absence of multivariable adjustment.

## 4 Discussion

This study provides real-world insights into the effectiveness and tolerability of Galcanezumab, Erenumab, and Rimegepant among Egyptian migraine patients, supporting findings from clinical trials and other real-world studies while highlighting their role in routine clinical practice.

The study population consisted predominantly of patients with chronic migraine and a high baseline disease burden, reflecting the severity of cases usually encountered in specialized clinical settings. The mean age was 21.9 years, and the majority were female, consistent with known migraine demographics (14, 15). Assessment of comorbid medical conditions showed high prevalence of psychiatric comorbidities, particularly anxiety and depression, which aligns with numerous studies that established a strong association between migraine and depression (16, 17) and prompts screening for psychiatric comorbidities in migraine management.

We observed ≥50% response rates of 65% for Erenumab,71% for both Galcanezumab, and Rimegepant, which is higher than those reported in pivotal clinical trials such as STRIVE (9), REGAIN (18), and phase 2/3 clinical trial evaluating oral Rimegepant for migraine prevention (19). Similarly, the average reduction in monthly migraine days (MMDs) was more pronounced in our population compared to trials. For example, Rimegepant achieved 9 days median reduction versus 4.3 days in the trial by Croop et al. (19). Erenumab and Galcanezumab showed 13- and 9.5-days median reduction, respectively—both substantially higher than reductions in pivotal trials (9, 18).

Our results corroborate real-world data from various countries including Japan, Korea, Italy, and the United States (20–23) which reported similar or slightly lower response rates and reductions in MMDs. Effectiveness was similar in all three treatment groups in our study, similar to the findings reported by Mahon et al. (24). Nonetheless, differences between the three medications in our study should not be interpreted as a comparison between them since there were significant baseline differences, and the study was not powered to address thus. Furthermore, there is no control group to compare to. Discontinuation cause among our cohort was primarily due to lack of improvement (20%) or substantial clinical improvement (12.5%) or cost-related factors. These findings are consistent with previous real-world studies reporting discontinuation due to both insufficient response and clinical improvement (25–27). Variability in discontinuation rates across studies likely reflects differences in healthcare systems, medication availability, affordability, insurance coverage and patient populations. These challenges underscore the importance of individualized care and patient education to improve long-term treatment access and adherence.

Among those who discontinued treatment, 42% experienced relapses, while 21% maintained their improvement. This is consistent with a study that assessed the early outcomes of migraine after Erenumab discontinuation and reported that 44% of patients experienced relapse within 1 to 4 weeks after discontinuation and another observational study conducted in Italy that reported relapse rate of 33.3% at three months follow up, and 41.7% at six months follow up (28, 29).

The observed relapse rates following treatment discontinuation suggests that underlying disease activity may persist despite clinical improvement and prompts the importance of careful consideration of treatment discontinuation strategies, highlighting that sustained benefit may require ongoing therapy especially in the chronic resistant migraine population.

While one of the initial aims of the study was to identify predictors of relapse following treatment discontinuation, the limited number of patients who experienced relapse precluded meaningful statistical analysis. Exploratory analyses suggested that treatment adherence and insurance coverage were associated with more favorable response patterns, while medication overuse headache showed a trend toward reduced response, although not statistically significant. These findings should be interpreted cautiously given the absence of multivariable adjustment.

Previous studies have identified factors such as lower baseline headache burden and fewer prior preventive treatment failures as being associated with better response (23, 30, 31). While similar trends were not observed in our cohort, this may be related to differences in sample size, population characteristics, and study design. Overall, these findings highlight the multifactorial nature of treatment response and the need for individualized approaches.

### 4.1 Limitations

This study was limited by the modest sample size that may limit the generalizability of results. Baseline imbalances in disease severity between treatment groups prevented comparative analysis of outcomes. The sample size calculation was based on within-patient change and was not specifically powered for between-group comparisons. Additionally, the relatively short follow-up period limits conclusions on long-term effectiveness and sustainability of treatment response.

### 4.2 Conclusion

This study provides real-world insights into the use of anti-CGRP therapies among migraine patients in Egypt, a region underrepresented in migraine research. The findings demonstrate preliminary descriptive evidence of clinically meaningful improvements across multiple outcome measures, along with generally favorable tolerability profiles in this cohort of severe and resistant migraine patients.

The study also highlights important aspects of treatment utilization, including adherence patterns and barriers to access, offering a comprehensive overview of anti-CGRP therapy in routine clinical practice. The findings require confirmation in larger, controlled, and adequately powered studies to better characterize factors associated with treatment response.

## Data Availability

The raw data supporting the conclusions of this article will be made available upon request to the corresponding author.

## Footnotes

1
www.ncss.com/software/pass/
